# Designing Clinical Trials for Assessing the Effectiveness of Interventions for Tinnitus

**DOI:** 10.1177/2331216517736689

**Published:** 2017-10-27

**Authors:** Deborah A. Hall

**Affiliations:** 1 Otology and Hearing Group, Division of Clinical Neuroscience, School of Medicine, University of Nottingham, UK; 2 Institute for Health Research (NIHR) Nottingham Biomedical Research Centre, UK; 3 Nottingham University Hospitals NHS Trust, Queen’s Medical Centre, UK

**Keywords:** heterogeneity, outcomes assessment, clinical trial, synthesis, population characteristics

## Abstract

In the face of finite resources, allocations of research and health-care funding are dependent upon high-quality evidence. Historically, tinnitus has been the poor cousin of hearing science, with low-quality clinical research providing unreliable estimates of effect and with devices marketed for tinnitus without strong evidence for those product claims. However, the tinnitus field is changing. Key opinion leaders have recently made calls to the field to improve the design, implementation, and reporting of clinical trials, and there is growing intersectoral collaboration. The Tonndorf Lecture presented at the 1st World Tinnitus Congress and the 12th International Tinnitus Seminar in Warsaw, Poland, provided an opportunity to reflect on the present and future progress of tinnitus research and treatment and what is needed for the field to achieve success. The content of that lecture is summarized in this article. The main debate concerns the selection and reporting of outcomes in clinical trials of tinnitus. Comprehensive reviews of the literature confirm the diversity of the personal impact of tinnitus and illustrate a lack of consensus in what aspects of tinnitus should be assessed and reported in a clinical trial. An innovative project is described which engages the global tinnitus community (patients and professionals alike) in working together. This project seeks to improve future tinnitus research by creating an evidence-based consensus about minimum reporting standards for outcomes in clinical trials of a tinnitus intervention. The output will be a core set of important and critical outcomes to be measured and reported in *all* clinical trials.

## Introduction

Every patient with tinnitus presents with a complex array of symptoms and functional impacts which reflects their own personal experience. The need for effective management options that cope with this heterogeneity in the clinical population has been widely recognized for many decades. For example, in his 1999 Tonndorf Lecture on the use of science to find successful tinnitus treatments, Richard Tyler (1999) looked ahead to a future in which a persuasive tinnitus treatment would be one that shows a large treatment effect, could be generalized across patients and clinicians, and would be specific and credible. Wide variability within individual patients tested over repeated assessments and large differences between patients allocated to a treatment group contribute to small overall treatment effects and lack of replicability of treatment-related findings across studies. Moreover, progress has generally been hampered by low quality in standards of design, conduct, and reporting of intervention trials, introducing an unacceptable risk of bias.

This situation thwarts attempts to make useful recommendations and practical guidelines for family medicine and primary health-care practitioners. Although there are a number of good practice guidelines for tinnitus (recently reviewed in Fuller et al., 2017), many therapeutic options are without evidence for their effectiveness. Furthermore, several recent systematic reviews evaluating the therapeutic benefits of specific interventions for tinnitus and published by Cochrane have shown that reporting is still flawed by poor methodology and poor reporting (e.g., Hilton, Zimmermann, & Hunt, 2013; Hoare, Edmondson-Jones, Sereda, Akeroyd, & Hall, 2014; Person, Puga, da Silva, & Torloni, 2016). Systematic reviews provide the highest level of evidence for treatment effectiveness, but rely on randomized controlled trials (RCTs) with a justified sample size and benefit from consistent use and reporting of common outcomes across studies. Indeed, a common conclusion for Cochrane reviews of tinnitus interventions is that “more high quality research is needed” because findings are inconclusive.

The Consolidated Standards of Reporting Trials (CONSORT) group provides perhaps the most well-known guidelines for solving problems arising from inadequate reporting of RCTs (CONSORT, 2017), and it has been endorsed by prominent general medical journals, many specialty medical journals, and leading editorial organizations. The CONSORT statement is an evidence-based minimum set of recommendations which provide a standard way for authors to prepare reports of trial findings (see Moher et al., 2010 for further details). The statement comprises (a) a 25-item checklist which can be followed to help report how the trial was designed, analyzed, and interpreted and (b) a flow diagram which helps to clearly illustrate how all participants progressed through the trial (including those who were screened but not randomized and those who withdrew and did not complete). The CONSORT statement seeks to facilitate authors’ complete and transparent reporting and aid their critical appraisal and interpretation. However, it does not appear to have had widespread uptake within the tinnitus community. To illustrate this point, a search, conducted using the U.S. National Library of Medicine National Institutes of Health PubMed database (on September 17, 2017), revealed only two publications (i.e., Hoare, Pierzycki, Thomas, McAlpine, & Hall, 2013; Stein et al., 2016) which contained the term “CONSORT” out of 11,372 possible articles on “tinnitus,” when these two search terms were cospecified to be present in any field.

Comparable tinnitus-centered statements have been around since the 1990s, and many of these recommendations are applicable to a range of trial designs, not just RCTs; they can even apply to the reporting of retrospective studies (Londero & Hall, 2017). In our recent Opinion article, we pooled together relevant concluding remarks or recommendations that had been taken from numerous review articles published by health-care and research leaders across the tinnitus community. Looking specifically at those comments about clinical trial outcomes in tinnitus, we noted that many of the authors repeat the same sort of advice. This indicates to us that these recommendations have probably not yet been very successful in transforming standards in the tinnitus field. As André Gide (French author, 1869–1951) said: “Everything that needs to be said has already been said. But since no one was listening, everything must be said again.”

## A Quest to Create a Legacy

An evidence-based hearing health-care system uses current best scientific evidence about what works best in making decisions about the care of the individual patient. To create that evidence, investigators test out interventions in clinical trials to make sure they work and are safe for patients. This is achieved by measuring “outcomes.” “Outcomes” refer collectively to those aspects of the condition that are chosen to assess how well the treatment has worked and the corresponding instruments for measuring them. Hence, outcomes have two facets. The first facet is the *outcome domain*, and this is defined as a complaint of tinnitus that is a distinct theoretical construct. Examples include how loud or how emotionally distressing a patient may find his or her tinnitus. The second facet is the *outcome instrument*, and this is defined as a tool used to assess and quantify the outcome domain. Outcomes can include aspects of the tinnitus sensation itself as well as the reactions to the tinnitus. Ideally, the primary outcome domains assessed in a clinical trial should be of importance to patients as well as health professionals, and outcome instruments should be reliable, validated, and responsive to treatment-related change. In any piece of clinical research, selecting and reporting outcomes are perhaps the most critical aspects. William Noble (2001, p. 20) perfectly summarized the importance of this issue when he wrote: “Critical to any form of treatment for tinnitus is the reliance placed on measures to assess the effectiveness of the intervention.” At worst, a study may fail because the outcome is inappropriate, not because the treatment is ineffective.

Over recent years, our work has focused on the issues of outcome domains and outcome instruments, specifically seeking to establish an evidence-based consensus about minimum reporting standards for outcomes in clinical trials that are evaluating any tinnitus intervention. To aid in this effort, a call was made to invite tinnitus experts representing different disciplines, different centers, and different countries to work together toward more consistent, evidence-based outcomes (Hall et al., 2015a). It is hoped that by actively seeking to engage directly with the international, multidisciplinary community in discussion, in research projects, and in consensus building, we can collectively create a set of research recommendations about minimum reporting standards that will be sufficiently influential for others to adopt of those ideas and recommendations into practice. This approach should enhance the likelihood of adoption of any recommendations, more so than simply relying on conventional dissemination channels, such as conference presentations and journal publications.

Harnessing the international community to collectively work toward solving some of these challenges is particularly important because unlike almost any other field of hearing health care, tinnitus is a topic of special interest to general practitioners; ear, nose, and throat physicians; audiologists; psychologists; neurologists; radiologists; and psychiatrists, as well as academic researchers and commercial representatives from the medical device and pharmaceutical sectors (Hall et al., 2011). A worthwhile legacy for tinnitus research would be to agree on a common conceptual framework and language for outcomes that is accessible and relevant to all relevant stakeholder groups.

## Trial Design Matters

In their Commentary on the status of systematic reviews, Clarke and Williamson (2016) noted that one of the greatest barriers to comparing, contrasting, and combining the findings of the existing research studies is the inconsistent use of outcome measures from one study to another. The field of tinnitus is no exception. For example, we found 133 different outcome instruments in use across clinical trials in tinnitus (Hall et al., 2016) and the existence of at least 29 different questionnaires that can be completed by patients to quantify an individual’s tinnitus symptoms (Haider, Fackrell, Kennedy, & Hall, 2016).

Which one(s) should be recommended for measuring treatment effects? Current advice is somewhat contradictory. For example, a small but influential group of tinnitus experts produced a statement that encompassed recommendations about the choice of treatment outcome measurement instruments (Langguth et al., 2007). The statement was that one standardized questionnaire should be used to measure treatment-related outcomes in all therapeutic trials, which is validated in many languages and in many cultural and socioeconomic groups. Recommendations at the time were for one of the following: Tinnitus Handicap Inventory, Tinnitus Handicap Questionnaire, Tinnitus Reaction Questionnaire, or the Tinnitus Questionnaire. However, the recommendations were not based on any review of the statistical performance of these instruments. Much less cited, perhaps because it is less well known, is a systematic review of the psychometric properties of these four questionnaires (Kamalski, Hoekstra, van Zanten, Grolman, & Rovers, 2010). Based on their findings, the authors called into question the validity, reliability, and responsiveness of these instruments for assessing treatment-related change (see also Fackrell, Hall, Barry, & Hoare, 2014). They recommended that more work be done before final conclusions are drawn regarding the utility of these specific questionnaires in future clinical studies. The American Academy of Otolaryngology Head and Neck Surgery (AAO-HNS) has echoed this research need. Following the publication of its clinical guideline of evidence-based recommendations for managing tinnitus (Tunkel et al., 2014), the organization also published a long list of research needs. Recommendations reiterated the need for further research to determine which instrument is most useful for assessing relevant treatment effects and also promoted the inclusion of a generic quality-of-life measure into clinical trials of tinnitus interventions to assess the net impact of any treatment-related benefits and harms (AAO-HNS, 2017). As a general rule, questionnaire instruments that successfully measure therapeutic benefit in different situations tend to be those with good statistical properties that enable the clinician or investigator to interpret specific complaints rather than those measuring a multidimensional health construct (Prinsen et al., 2016).

**Figure 1.**
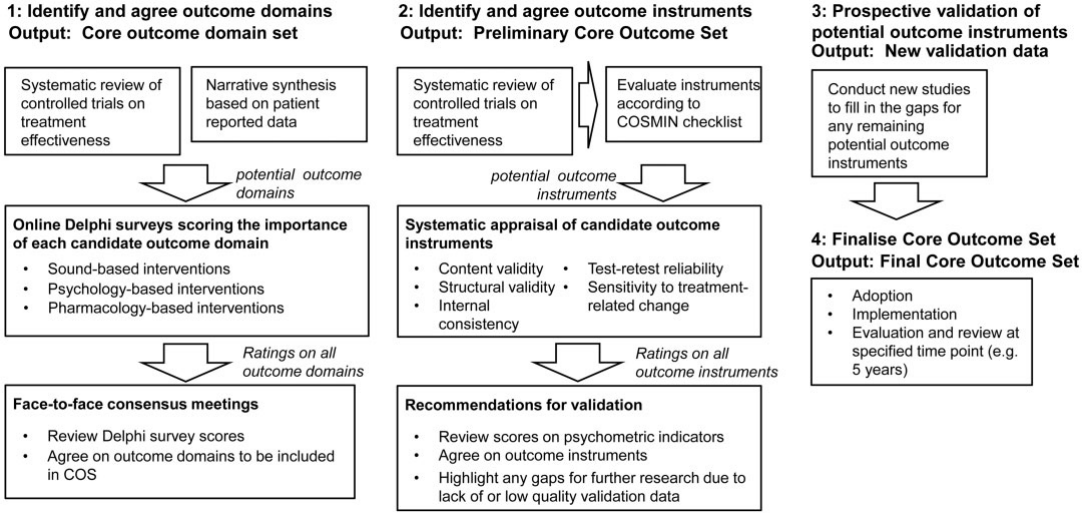
An updated summary of the roadmap to establish an international standard for outcome assessment and reporting in early phase clinical trials of tinnitus, adapted from Hall et al. (2015a). In this scheme, an outcome domain is a complaint of tinnitus that is a distinct theoretical construct, and an outcome instrument is a tool used to assess and quantify that outcome domain. Instruments are not limited to questionnaires but can include other tools such as clinician-administered tests.

In Europe, a TINnitus NETwork (TINNET) consortium has been formed to address this research need by establishing standards for clinical trials in tinnitus. The consortium was established through an EU COST Action funding (BM1306, 2014–2018) and supports the COMiT (Core Outcome Measures in Tinnitus) initiative—a network of partners interested in outcomes. As already indicated, intentions to compare, contrast, and combine the findings of existing research studies are often thwarted by inconsistencies in the outcomes that were measured and reported in the individual studies. This, in turn, makes it difficult for the tinnitus community to make informed decisions and choices about effective health and social care. One solution would be for tinnitus trials to measure and report a standardized set of outcomes, which would then also be used in systematic reviews (Clarke & Williamson, 2016). We published a roadmap that set out the research process by which we hope to achieve an evidence-based consensus on a standardized collection of tinnitus-related outcomes (Hall et al., 2015a). An updated summary of that roadmap is given in Figure 1. The roadmap was accompanied with a call inviting tinnitus experts to engage with the COMiT initiative (Hall et al., 2015a); the group currently comprises 46 tinnitus experts from across 17 countries (TINNET, 2017).

## Standardizing Outcome Reporting

The roadmap for establishing standards for outcome measurements in clinical trials for tinnitus starts with outcome domains (Figure 1). The purpose of Step 1 is to identify and agree on a core set of outcome domains where all those outcome domains will be measured and reported in all clinical trials evaluating an intervention for tinnitus. Step 1 began with two reviews of the literature: one to understand what tinnitus-related complaints are relevant to professionals as reported in clinical trials (Hall et al., 2016; Hall, Szczepek, Kennedy, & Haider, 2015b) and one to understand what tinnitus-related complaints are relevant to patients (Haider et al., 2016).

### Professional Perspectives on Tinnitus-Related Complaints

In the review of research relevant to professionals, clinical trials of tinnitus were identified by searching four of the major electronic databases of scientific publications, three international clinical trial registries, and the Cochrane Database of Systematic Reviews (Hall et al., 2016). From 2,077 articles identified, 228 met our eligibility criteria: (a) published from July 2006 to March 2015; (b) enrolling adults aged 18 years or older; (c) participants reported tinnitus as a primary complaint; (d) RCT design, before and after the study, non-RCT, case–control study, or cohort study; (e) sample size of at least 20; and (f) published in English.

According to the reporting of the study design, 61 different outcome domains were identified spanning seven categories (tinnitus percept, impact of tinnitus, co-occurring complaints, quality of life, body structures and function, treatment-related outcomes, and unclear or not defined). This heterogeneity across studies is symptomatic of the lack of consensus among tinnitus experts. Most common were tinnitus loudness (10%, 112 of 1,084) and the effects of tinnitus on feelings of distress (5%, 51 of 1,084). While the majority of outcome domains were related to therapeutic benefit, harms were also assessed and reported (7%, 79 of 1,084). Typically, reporting was described as “safety” or “side effects” (4% and 1%, respectively). Of note, in 50% of cases (539 of 1,084), we observed that investigators did *not* clearly report the outcome domain of interest. Many times it was not mentioned at all, while other times it was merely described as “tinnitus handicap” or “tinnitus severity.” Tinnitus handicap did not meet the COMiT initiative’s requirement for an outcome domain because it refers generally to any disadvantage that limits or prevents the fulfilment of a person’s role that is normal. It is not a distinct theoretical construct. Equally, tinnitus severity did not meet our requirement because it refers to the magnitude of the burden of tinnitus on the patient. It does not define the complaint itself.

### Patient Perspectives on Relevant Tinnitus-Related Complaints

The main objective of the patient-centric review was to identify what adults with tinnitus and their significant others report as problems in their everyday lives caused by tinnitus (Haider et al., 2016; Hall et al., 2017). Studies were identified in which participants were enrolled because tinnitus was their primary complaint. To do this, electronic searches were conducted in PubMed, Embase, CINAHL, as well as grey literature sources to identify publications from January 1980 to June 2015. A manual search of seven relevant journals then updated the search to February 2017. Of the 3,638 titles identified overall, 81 records (reporting 83 studies) met our inclusion criteria and were taken through to data collection representing 15,902 study participants with tinnitus. Coders collated all reported generic and tinnitus-specific complaints, which were then synthesized into a list of items each describing theoretically distinct constructs. Overall, there were 42 discrete unidimensional patient-reported complaints. These spanned eight categories (negative attributes of the tinnitus percept, physical health problems, functional difficulties due to the tinnitus, emotional complaints associated with tinnitus-related distress, negative thoughts about tinnitus, general mood states such as anxiety and depression, and aspects of quality of life). Most common were the effects of tinnitus on feelings of distress and sleep difficulties, but every complaint is a potential outcome domain that could be measured in a clinical trial to assess treatment-related change.

These unpublished findings emphasize the vast array of personal experiences associated with tinnitus. All these complaints are candidates to be considered as outcome domains used to assess whether a treatment has worked. Examples of these are reported and described in Table 1. Considerable work has gone into selecting only those outcome domains which refer to a conceptually distinct element of tinnitus, excluding broad overarching constructs (such as “Quality of Life” or “Cognitive difficulties”; Fackrell et al., 2017). In addition, outcome domain names and plain language descriptions have been coproduced with 16 people who have tinnitus and 5 clinical experts (Fackrell et al., 2017). The list given in Table 1 is extensive. Clearly, it is not feasible to assess so many outcome domains in every clinical trial evaluating an intervention for tinnitus. The list therefore needs to be reduced to a minimum reporting set, which represents a small number of outcome domains that the tinnitus community agree are critically important for assessing therapeutic outcome and so should always be assessed and reported.

**Table 1.** Table of Outcome Domain Categories and Tinnitus-Related Outcome Domains Informed by the Evidence Collected From the Literature.

Category	Tinnitus-related outcome domain	Description of the concept
Behavior	Behavior	A difference in the way somebody behaves or acts, particularly in response to a particular event or situation
Body structures and functions	Brain structure	Looking at different parts of the brain
	Fat metabolism	An important process for creating energy in the body and for building new cells
	Gene expression	The way in which particular genes generate proteins and other complex molecules that have an impact on health or disease
	Neural activity	Activity of cells in the brain
	Neuroendocrine hormones	Specific hormones that can affect physical and mental states
	Oxidative stress	An imbalance between harmful chemicals and the ability of the body to counteract or “detoxify” their harmful effects
Cognition (thought processes)	Ability to ignore	Ability to continue as normal as if tinnitus were not there
	Concentration	Ability to keep your attention focused
	Confusion	Being unable to think clearly, either in general or specifically associated with your tinnitus
	Tinnitus-related thoughts	Thoughts about tinnitus
Coping and acceptance	Acceptance of tinnitus	Recognizing that tinnitus is a part of your life without having a negative reaction to it
	Coping	Ability to deal with or handle tinnitus (includes the use of techniques)
Effects of tinnitus on hearing	Conversations	Effect of tinnitus (not hearing loss) on ability to listen, understand, and take part in conversations
	Listening	Effect of tinnitus on ability to understand somebody talking (e.g., TV and radio)
Emotions	Anger	Feelings of aggression, either in general or specifically associated with your tinnitus
	Annoyance	Noticing the sound of tinnitus is there and it feels like a nuisance
	Anxiety	Feeling of unease, either in general or specifically associated with your tinnitus
	Depressive symptoms	Feelings of low mood, hopelessness, or lack of interest, either in general or specifically associated with your tinnitus
	Distress from bodily sensations	How physical feelings or pain cause emotional distress
	Fear	Afraid tinnitus will have an impact on physical and mental health, now and in the future, or will become worse
	Helplessness (lack of control)	Feeling despair about being unable to control or manage tinnitus
	Irritable	Having a tendency to easily feel tense, on edge or agitated because of your tinnitus
	Joyful	General ability to feel pleasure and happiness
	Mood	General sense of well-being, ranging from feeling very low or negative to very positive
	Nervous	Feeling agitated, uneasy, or apprehensive about or because of your tinnitus
	Upset	Feeling unhappy or disappointed because of your tinnitus
	Worries/concerns	To feel troubled and uncertain, either in general or specifically associated with your tinnitus
Factors related to the treatment being tested	Adverse reaction	Any bad or unexpected thing that happens during the time a treatment is being tested and are thought to be a result of that treatment being tested
	Pharmacokinetics	The way the body absorbs, distributes, and gets rid of a drug
	Treatment satisfaction	How the treatment meets your expectations or how pleased you are after receiving the treatment
	Withdrawal from treatment in the clinical trial	How many people stopped using the treatment during the trial
Health-related quality of life	Impact on individual activities	Effect of tinnitus on your choice to engage in your individual interests or tasks (e.g., driving, reading, listening to music, or watching TV). Not group activities
	Impact on relationships	Effect of tinnitus on relationships with family and friends
	Impact on social life	Effect of tinnitus on the ability to take part fully in a group social gathering (e.g., at a restaurant, at the park, or at a party)
	Impact on work	Effect of tinnitus on your ability to carry out work tasks or job roles
	Sexual difficulties	Difficulty experienced by an individual or a couple during any stage of sexual activity
Negative thoughts	Catastrophizing	An exaggerated negative way of thinking about tinnitus or tinnitus-related symptoms
	Irrational beliefs	Illogical conclusions or beliefs about your tinnitus
	Negative thoughts/beliefs	Thinking tinnitus will affect you in a negative way (e.g., thinking that tinnitus is never going to get better or that it would be dreadful if these noises never went away)
	Suicidal thoughts	Thoughts about committing suicide
Perceptions of the tinnitus sound	Tinnitus awareness	Noticing the sound of tinnitus is there
	Tinnitus intrusiveness	Noticing the sound of tinnitus is there and it is invading your life or your personal space
	Tinnitus location	Where the tinnitus sound is heard (e.g., left ear, right ear, both ears, or in the head)
	Tinnitus loudness	How loud your tinnitus sounds
	Tinnitus pitch	Whether your tinnitus has a note-like quality, for example, high pitch like whistling or low pitch like humming
	Tinnitus quality	What type of sound is heard (e.g., hissing, buzzing, ringing, whistling, etc.) and whether it is constant or fluctuating (e.g., stable or wobbling)
	Tinnitus unpleasantness	Tinnitus making you feel disagreeable or uncomfortable
Physical health	Ability to relax	Ability to release physical and mental tension
	Active myofascial trigger points	A particular area in a muscle that is excessively sensitive to pressure
	Bodily complaints	Headaches, nausea, ear pressure, or muscle tension associated with tinnitus
	Difficulties getting to sleep	Problems in getting to sleep or problems in getting back to sleep after waking up at night
	Feeling tired	Lacking energy
	Ill health	Generally feeling unwell
	Loss of appetite	Loss of natural desire to eat
	Neck mobility	Ability to move the neck freely and easily
	Neck pain	Unpleasant physical sensation in the neck
	Pain	A feeling of noticeable discomfort in a particular part of the body, associated with tinnitus
	Quality of sleep	Getting the right amount of undisturbed sleep for you that leaves you feeling refreshed and rested
State of mind	Change in sense of self	A change in the way you see yourself (e.g., feeling insecure or lacking in confidence)
	Loss of peace	Loss of sense of calm
	Sense of control	Whether or not you feel you have a choice in how to manage the impact of tinnitus and feelings caused by tinnitus
Support and knowledge	Need for knowledge	Wanting to gain knowledge and understanding about the facts related to tinnitus
	Lack of perceived support “nobody understanding experience”	Feeling a lack of support from others due to their limited awareness and understanding of tinnitus (e.g., friends, family, health-care worker, work colleagues)
	Seeking support	Motivation to talk about tinnitus with others, seeking advice, and support (e.g., friends and family, health-care worker, support group)
	Support from family, friends, or health-care workers	Feeling that friends, family, or health-care workers are there for you.

*Note.* Descriptions of each concept were coproduced with people with lived experience of tinnitus in order to ensure that the explanation and meaning is accessible to a range of interested parties, irrespective of their technical expertise in tinnitus health care.

### Minimum Set of Outcome Domains for Clinical Trials of Sound-, Psychology-, and Pharmacology-Based Tinnitus Interventions

**Figure 2.**
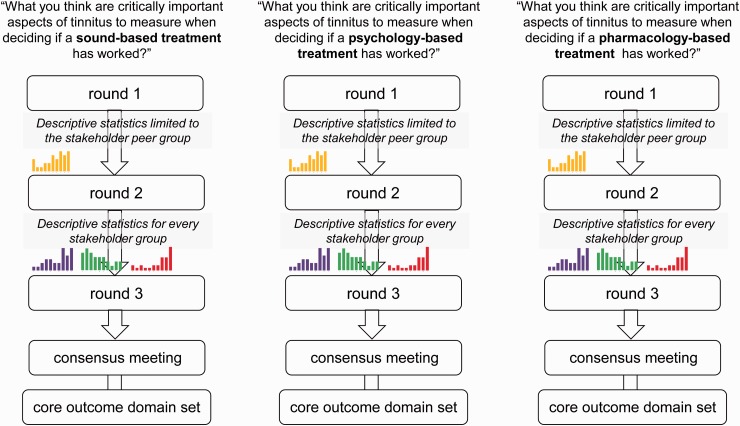
A schematic diagram of the online Delphi process, including Rounds 1 to 3 and the face-to-face consensus meetings. The colored histograms represent the planned graphical format of the results from the previous round. Single (yellow) histogram represents results for the peer stakeholder group. Purple, green, and red histograms represent results for each relevant stakeholder group (peer and otherwise).

To achieve this minimum set of outcome domains, the final part of Step 1 in the roadmap is to reduce the “long list” described earlier through a consensus approach involving all key stakeholders (professionals and patients alike). After discussion with tinnitus health-care practitioners and commercial representatives, the COMiT initiative decided that specific discussions were needed around the major therapeutic approaches for tinnitus (namely, sound-, psychology-, and pharmacology-based interventions) because they do not necessarily target the same tinnitus-related complaints (Fackrell et al., 2017). Our study design therefore includes three separate surveys, each based on an online Delphi survey method and each recruiting experts in that particular therapeutic strategy (Figure 2). This study has been given a favorable ethics opinion by the West Midlands Solihull Research Ethics Committee (ref: 17/WM/0095) and is almost completed. The outcome will be three core outcome domain sets; one for each intervention category. The Delphi survey method is suited to explore areas where controversy, debate, or a lack of clarity exist (Iqbal & Pipon-Young, 2009). It has been used to determine the range of opinions on specific matters and to achieve consensus on disputed topics. Although there is no gold standard for how the Delphi method is applied, its distinct characteristics are the following:

1. It recruits a group of participants specially selected for their particular expertise on a topic;
2. It is often conducted across a series of two or more sequential questionnaires known as “rounds,” with Round 1 enabling participants to nominate salient issues (in this case, candidate outcome domains that were missing from the “long list”);
3. It has an evaluation phase where participants are provided with a summary of the stakeholder responses and asked to reevaluate their original responses; and
4. It is interested in the formation of consensus, often defined as the number of participants agreeing with each other on questionnaire items (Iqbal & Pipon-Young, 2009).

By making the surveys accessible online and through calls to participate via a range of dissemination channels (publications, conferences, patient organizations, and social media), 308 professionals and 366 patients from across the world have participated in Round 1 of the Delphi surveys (including 91 from the United States and Canada and 582 from Europe). Stakeholder groups are people with lived experience of tinnitus, health-care practitioners, clinical researchers, commercial representatives and funders, as well as journal editors. In Round 1, we asked participants to think about each one of the distinct outcome domains listed in Table 1. Participants scored each outcome domain using the GRADE scale of 1 to 9, where 1 represents *least important* and 9 represents *most important* (Guyatt et al., 2011). Selecting response options 1 to 3 indicates that the domain is *not important*, while 7 to 9 indicates that the domain is *critically important* in deciding whether a tinnitus treatment is effective. Scores 4, 5, and 6 indicate the outcome domain is *important but not critical*. Participants could also tell us if we had missed anything, and these would be added as new candidate outcome domains in Round 2. In Rounds 2 and 3, all participants received the same list of outcomes with feedback tailored according to their stakeholder allocation. The purpose of Round 2 is to enable participants to reflect on their scores in light of the viewpoint of their stakeholder peers and to score the outcomes again (Figure 2). The purpose of Round 3 is to enable participants to reflect on their scores in light of the viewpoint of their stakeholder peers and all other stakeholder groups and to score the outcomes again (Figure 2). At the end of Round 3, if at least 70% of experts of the participants in each stakeholder group score 7 to 9 agree and fewer than 15% score 1 to 3, then we will recommend that outcome domain for the intervention-specific Core Outcome Set (cf. Williamson et al., 2012).

Consensus meetings, planned for September and October 2017, will agree on outcome domains to be included in the Core Outcome Set. There will be one meeting for each intervention category and a representative subset of participants will discuss and then vote on each outcome domain as “in” or “out.” Once we have identified a core outcome domain set, the next step in our roadmap is about selecting “how” and “when” to measure them. This corresponds to Step 2 in the roadmap (Figure 1). By “how” we mean what instrument. By “when” we mean is it a short-term change that should be seen immediately after the treatment, or is it a long-term change that will be sustained months and years after treatment. Again, these are decisions to be taken with consensus across the tinnitus community. Collectively, the “what,” “how,” and “when” is called a Core Outcome Set.

## Recommendations

The work conducted so far through the COMiT initiative has already enabled us to start making some evidence-based recommendations about reporting standards. In particular, 17 members met on March 16, 2016 during the first EU COST Action TINNET conference held in Nottingham to discuss the implications of the evidence base gathered as part of a recent systematic review of clinical trials of tinnitus interventions for adults with tinnitus (Hall et al., 2016; Hall et al., 2015b). From this evidence base, we highlighted a number of suggestions to the tinnitus community concerning the specification and reporting of outcomes in clinical trials (TINNET, 2016). The aim of our simple guidelines is to harmonize the reporting of clinical trial outcomes for tinnitus. These recommendations are intended not only for investigators’ designing and reporting trials but also for journal editors and journal reviewers who play an important role in the publication process.

### Prespecify the Primary Determinant(s) of Clinical Effectiveness

The simplest and most common trial design is where one primary outcome domain determines whether or not the intervention is judged to be effective. For example, the main goal of an intervention may be to improve the quality of sleep. But sometimes an intervention can be intended to have a positive influence on more than one distinct tinnitus-related complaint (such as reducing tinnitus loudness, *and* tinnitus intrusiveness, *and* sense of control). In that scenario, investigators would therefore have a good motivation to measure all three outcomes in a trial. The default expectation would then be that all three outcomes should demonstrate a significant reduction in order to drive any conclusions about the therapeutic benefit. If there is no clear a priori proposal to treat the three outcomes differently, then if only one showed a significant reduction, there is a risk that this will be emphasized in the reporting, while the other two are downplayed. This is what CONSORT (2017) calls “risk of bias in outcome reporting.” And if such a trial was included in a systematic review, then this would present a source of potential methodological concern. To minimize outcome reporting bias, investigators should specify a priori which of the potential tinnitus-related complaints will determine the conclusion about treatment effectiveness, and how the findings will be interpreted. Any number of additional outcomes can be defined either as secondary outcomes or exploratory outcomes, but these would not be expected to drive any conclusions about therapeutic benefit.

### Describe “What,” “How,” and “When”

For reporting the primary outcome(s) at least, the COMiT initiative has endorsed a simple formula that considers *what* is measured, *how* it is measured, and *when* it is measured. Table 2 provides several worked examples.

**Table 2.** Worked Examples of Outcome Reporting (What, How, and When).

	Worked examples of outcome reporting (what, how, and when)
1	“Functional impact of tinnitus, measuring using the Tinnitus Functional Index (Meikle et al. Ear Hear. 2012; 33(2):153-76) at 12 weeks after hearing aid fitting.”
2	“Psychological impact of tinnitus, measured using an Italian adaptation of the Tinnitus Handicap Inventory (Monzani et al. Acta Otorhinolaryngologica Italica. 2008;28(3):126-34) at 6 months after the start of a 12-week stepped-care programme.”

*Note.* These are examples of the recommended reporting format. They do not constitute an endorsement of the outcome measurement instruments.

***What:*** For trials of clinical efficacy and effectiveness, investigators should clearly specify what they expect their intervention to change. Examples include “how loud the tinnitus is perceived” or “how distressed the person feels by their tinnitus.” These specific dimensions or domains of tinnitus-related complaint define the clinical trial outcome.

***How:*** For each outcome, investigators should clearly specify how that outcome domain will be measured. In other words, which specific instrument will be used to measure change in that tinnitus-related complaint, and preferably that instrument should have been evaluated for responsiveness to treatment-related change in the appropriate patient population because just because it is shown to be sensitive in one population does not necessarily mean it is sensitive in another (Fackrell, Hall, Barry, & Hoare, 2016). It is also preferable to provide some explanation about why that particular instrument was selected.

***When:*** For each outcome of interest, investigators should clearly specify when the outcome will be measured. In our review of tinnitus trials, we found this information particularly difficult to extract from published reports (Hall et al., 2016), and to avoid confusion, we suggest that it is preferable to describe the end point relative to the start (not the end) of the intervention period. This avoids any misinterpretation about the timescales between the pre- and postintervention assessments.

### Patient Harms Are Important Too

Cuervo and Clarke (2003) said that “Reporting harms may cause more trouble and discredit than the fame and glory associated with successful reporting of benefits (1). Reporting harms may cause more trouble and discredit than the fame and glory associated with successful reporting of benefits” (p. 66). Perhaps then it is no surprise that in our review of tinnitus trials we found very little published information about the negative effects of the intervention of interest (Hall et al., 2016). Yet, the purpose of a trial is to collect and appropriately report good and bad events and outcomes so that they may be compared across treatment groups (Ioannidis et al., 2004). In addition to providing reliable evidence on the beneficial effects of an intervention, it is just as important to provide reliable information about its harms. Harms can be thought of as the direct opposite of benefits and examples include withdrawals and adverse events. We highlight an extension of the CONSORT statement that gives guidance on reporting harms (Ioannidis et al., 2004). Harms is the preferred term over “safety” or “side effects.” This is because safety refers to substantive evidence for the absence of harm, not the absence of evidence of harm, while side effects imply that the harms are caused by the intervention. Yet this is not always known.

## Concluding Remarks

The recommendations endorsed by the COMiT initiative are consistent with international reporting guidelines aiming at positively influencing the quality of published research reports for RCTs. The CONSORT statement is extremely helpful (CONSORT, 2017; Moher et al., 2010). The CONSORT statement can also be used to guide reporting other trial designs, including parallel non-randomized trials and cross-over designs. Readers might find it useful to know that the EQUATOR network (Enhancing the QUAlity and Transparency Of health Research) maintains an up-to-date library of this and other reporting guidelines and toolkits for authors (www.equator-network.org).

The COMiT initiative is open to views from all stakeholders interested in the development of Core Outcome Sets for tinnitus. There is a strong passion and shared optimism for working together and engaging with tinnitus experts outside the European Union in order to ensure that our recommendations truly reflect an international consensus. We particularly encourage health-care practitioners and researchers from North America, Australasia, Asia, and Africa to act as a national advocate for the project and to help us spread the adoption and implementation of our recommendations as a model of good practice.
